# SARS in Cars: Carbon Dioxide Levels Provide a Simple Means to Assess Ventilation in Motor Vehicles

**DOI:** 10.20411/pai.v7i1.493

**Published:** 2022-02-02

**Authors:** Muhammed F. Haq, Jennifer L. Cadnum, Matthew Carlisle, Michelle T. Hecker, Curtis J. Donskey

**Affiliations:** 1 Research Service, Louis Stokes Cleveland VA Medical Center, Cleveland, Ohio; 2 Department of Infectious Diseases, MetroHealth Medical Center, Cleveland, Ohio; 3 Case Western Reserve University School of Medicine, Cleveland, Ohio; 4 Research, Education, and Clinical Center, Louis Stokes Cleveland VA Medical Center, Cleveland, Ohio

**Keywords:** Ventilation, carbon dioxide, transmission, aerosol, SARS-CoV-2, COVID-19

## Abstract

**Background:**

Poorly ventilated enclosed spaces pose a risk for airborne transmission of severe acute respiratory syndrome coronavirus 2 (SARS-CoV-2) and other respiratory viruses. Limited information is available on ventilation in motor vehicles under differing driving conditions.

**Methods:**

We conducted carbon dioxide measurements to assess ventilation in motor vehicles under varying driving conditions with 2 to 3 vehicle occupants. During routine driving, carbon dioxide produced by the breathing of vehicle occupants was measured inside 5 cars and a van under a variety of driving conditions with or without the ventilation fan on and with windows open or closed. Carbon dioxide readings above 800 parts per million (ppm) were considered an indicator of suboptimal ventilation.

**Results:**

Carbon dioxide levels remained below 800 ppm in all vehicles if the ventilation fan was on and/or the windows were open while parked or during city or highway driving. With the ventilation system set on non-recirculation mode, carbon dioxide levels rose above 800 ppm in all vehicles when the fan was off and the windows were closed while parked and during city driving, and in 2 of the 6 vehicles during highway driving. With the ventilation system set on recirculation mode, carbon dioxide rose above 800 ppm within 10 minutes in all vehicles tested.

**Conclusion:**

Carbon dioxide measurements could provide a practical and rapid method to assess ventilation in motor vehicles. Simple measures such as opening windows, turning on the fan, and avoiding the recirculation mode greatly improve ventilation.

## INTRODUCTION

Shared motor vehicle travel poses a risk for transmission of severe acute respiratory syndrome coronavirus 2 (SARS-CoV-2) and other respiratory viruses [1–4]. In an outbreak in China in January 2020, an infected source passenger in a bus was linked to multiple infections in fellow passengers, including individuals seated more than 2 meters away [4]. Recently, we reported 2 episodes of SARS-CoV-2 transmission from infected van drivers to multiple passengers despite masking and physical distancing [1]. With the heater operating, it was demonstrated that fluores-cent microspheres were transported by airflow more than 3 meters from the front to the back of the van [1].

In addition to masking and physical distancing, the Centers for Disease Control and Prevention (CDC) recommends that efforts be taken to improve ventilation in motor vehicles when possible by opening windows and setting the ventilation system on non-recirculation mode [3]. Although relatively little published information is available on ventilation in motor vehicles, there is evidence that ventilation and risk for viral transmission may vary considerably under different driving conditions and in different types of vehicles [5, 6]. To reduce the risk for transmission of respiratory viruses, there is a need for practical, inexpensive, and rapid tools to assess ventilation in motor vehicles.

Carbon dioxide levels are often used as an indicator of ventilation in occupied indoor environments [7–12]. The concentration of carbon dioxide in outdoor air is approximately 400 parts per million (PPM) versus approximately 40,000 ppm in exhaled breath [9]. Thus, carbon dioxide levels rise in occupied spaces that are poorly ventilated. The goal of the current study was to examine the use of carbon dioxide levels as a practical tool to assess ventilation in motor vehicles under varying driving conditions.

## METHODS

### Test vehicles

The test vehicles included 5 cars and a van. The cars included 3 compact cars (defined as between 100 to 109 cubic feet of combined passenger and cargo space) and 2 midsize cars (defined as between 110 and 119 cubic feet of combined passenger and cargo space). For the purposes of the study, the cars were numbered 1 through 5 and the make/model was not identified; car 1 was a midsize sedan, car 2 was a compact sports utility vehicle, car 3 was a midsize sedan, car 4 was a compact hatchback, and car 5 was a compact sports utility vehicle. The van had approximately 160 cubic feet of combined passenger and cargo space. The cars had air vents allowing air entry to the car interior through the front foot-wells and dashboard when the car was moving or when the ventilation fan was on. For each of the cars, air exits through rear vents providing a constant flow of air through the interior when driving or when the fan is on; the airflow is single pass unless on recirculation mode. The van had front and side air vents for air entry and rear vents for air exit.

### Carbon dioxide levels in the cars and a van under varying driving conditions

The MetroHealth Medical Center's Institutional Review Board determined that the study procedures were exempt. Consent was obtained from the drivers, but no identifying information was recorded, and the specific make and model of vehicles was not disclosed. Human experimentation guidelines of the institution were followed. The participants measured carbon dioxide levels in the cars and van when parked and under a variety of driving conditions during routine driving trips such as commuting to and from work or driving for personal business. For the cars, measurements were taken during trips when a driver and 1 passenger occupied the vehicle; for the van, measurements were taken when a driver and 2 passengers were in the vehicle. The carbon dioxide was naturally produced by the driver and passengers through respiration. The carbon dioxide levels were continuously monitored using an IAQ-MAX CO2 Monitor and Data Logger (CO2Meter, Inc) that was placed in the back seat of the car or in the middle section of the van; the monitor allows readings to be downloaded after completion of trips. In preliminary experiments, carbon dioxide readings were demonstrated to be similar when the monitor was placed in different locations within the vehicle. Based on recommendations from the CDC for ventilation benchmarks for buildings, carbon dioxide readings above 800 ppm were considered an indicator of suboptimal ventilation requiring intervention [13].

The drivers were asked to obtain carbon dioxide readings with the vehicle parked under different ventilation conditions. The ventilation conditions included fan off and windows closed, fan on and windows closed, and fan off and 1 front window fully opened. In preliminary experiments in 1 vehicle, the increase in carbon dioxide levels inside the vehicle was equivalent to the increase observed in a non-ventilated 41.7 m^3^ room (ie, >2,000 ppm within 15 minutes).

The drivers were asked to adjust the ventilation conditions in the vehicle during driving trips and to record the route traveled, ventilation settings, and the driving conditions. Before the start of each trip, the driver opened a front window for 5 minutes to adjust the carbon dioxide level to approximately 400 to 450 ppm. Preliminary experiments demonstrated that carbon dioxide levels within the vehicles rapidly equilibrated within 1 to 2 minutes to levels measured in outdoor air when 1 front window was opened either fully or partially (ie, 6 inches down) during highway or city driving, consistent with rapid inflow of outdoor air; when parked, carbon dioxide levels equilibrated within 5 minutes to levels measured in outdoor air with the window fully opened. The ventilation conditions included, 1) city driving with fan on and windows closed; 2) city driving with fan off and windows closed; 3) highway driving with fan off and windows closed; 4) highway driving with fan on and windows closed; and 5) city driving with fan off and 1 front window either partially or fully open. Driving routes were classified as city driving if the route included primarily city streets with a speed limit of 25 miles per hour with frequent stops. Highway driving was at a speed of 60 to 70 miles per hour. Driving with the window open served as a control for carbon dioxide levels in outdoor air. For each of these driving conditions, the ventilation system was set on non-recirculation mode. When the fan was on, the medium setting was chosen. The carbon dioxide readings were recorded at 1-minute intervals. Data was only included in the analysis if at least 10 minutes of readings were recorded for a given driving condition. For each vehicle, data was obtained for at least 2 separate driving episodes under each driving condition.

For 3 of the cars (cars 1 to 3), carbon dioxide measurements were also collected during highway driving for 20 to 25 minutes with the ventilation system set on recirculation mode. The rationale for this assessment was that the CDC recommends the ventilation system of motor vehicles be set on non-recirculation mode when multiple occupants are present [3].

### Air flow from the air vents

For the van and 2 of the cars (car 1 and car 2), air flow from the front air vents was measured under the different driving conditions using a mini thermo anemometer (EXTECH, AN100). The purpose of this assessment was to determine if differences in air flow from the vents would explain differences in carbon dioxide accumulation for 3 of the vehicles during highway driving with the fan off.

## RESULTS

### Carbon dioxide levels in parked vehicles under different ventilation conditions

**Figure 1.**
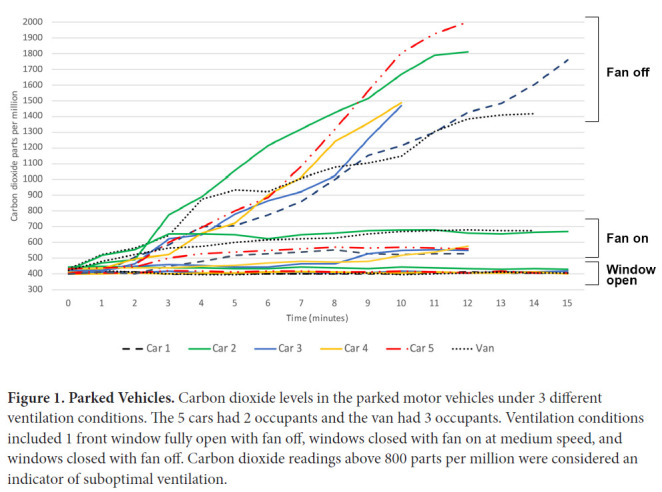
**Parked Vehicles.** Carbon dioxide levels in the parked motor vehicles under 3 different ventilation conditions. The 5 cars had 2 occupants and the van had 3 occupants. Ventilation conditions included 1 front window fully open with fan off, windows closed with fan on at medium speed, and windows closed with fan off. Carbon dioxide readings above 800 parts per million were considered an indicator of suboptimal ventilation.

Figure 1 shows the carbon dioxide levels in the 5 cars (2 occupants) and van (3 occupants) when parked under different ventilation conditions. With 1 front window open, the carbon dioxide levels remained approximately 400 ppm consistent with outdoor air. With the fan off and windows closed, the carbon dioxide levels rose gradually from approximately 400 ppm to greater than 1,300 ppm in all the vehicles. With the fan on and windows closed, the carbon dioxide levels rose to greater than 500 but remained below 700 ppm in all vehicles (peak carbon dioxide levels ranged from 529 to 681).

### Carbon dioxide levels in cars with ventilation on recirculation mode

**Figure 2.**
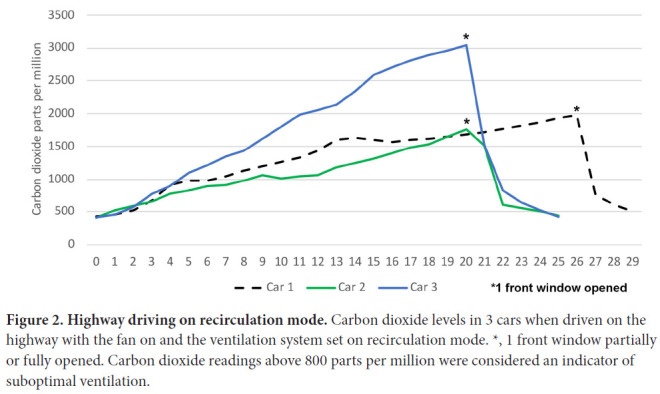
**Highway driving on recirculation mode.** Carbon dioxide levels in 3 cars when driven on the highway with the fan on and the ventilation system set on recirculation mode. *, 1 front window partially or fully opened. Carbon dioxide readings above 800 parts per million were considered an indicator of suboptimal ventilation.

Figure 2 shows carbon dioxide levels for cars 1 to 3 when driven on the highway with the ventilation system set on recirculation mode. The carbon dioxide levels rose gradually in all 3 cars peaking at 1760 ppm or higher after 20 to 25 minutes. The carbon dioxide levels rapidly decreased to below 500 ppm when 1 front window was opened while driving. Carbon dioxide levels increased similarly in each of the vehicles when driven in the city at 25 to 35 miles per hour with frequent stops (data not shown).

### Carbon dioxide levels in cars during car trips under varying driving conditions

**Figure 3.**
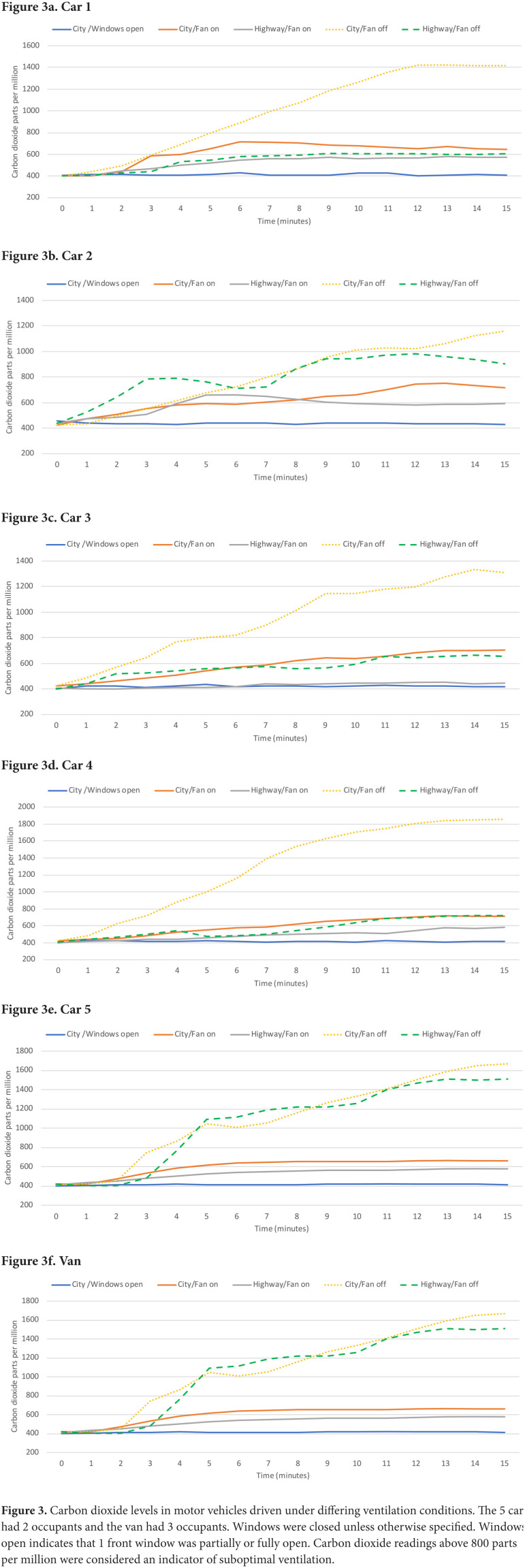
Carbon dioxide levels in motor vehicles driven under differing ventilation conditions. The 5 cars had 2 occupants and the van had 3 occupants. Windows were closed unless otherwise specified. Windows open indicates that 1 front window was partially or fully open. Carbon dioxide readings above 800 parts per million were considered an indicator of suboptimal ventilation.

Figure 3 shows the carbon dioxide levels in the 5 cars (2 occupants) and van (3 occupants) under differing driving conditions. With 1 front window open while driving in the city, the carbon dioxide levels remained approximately 400 ppm consistent with outdoor air. With the fan off and windows closed while driving in the city, the carbon dioxide levels rose gradually from approximately 400 ppm to greater than 1,100 ppm in all the vehicles. With the fan off and windows closed while driving on the highway, the carbon dioxide level rose but remained below 800 ppm in all vehicles except car 2 and the van. With the fan on and windows closed, the carbon dioxide levels remained below 800 ppm in all vehicles during highway and city driving.

### Air flow from the front air vents of 3 vehicles

**Figure 4.**
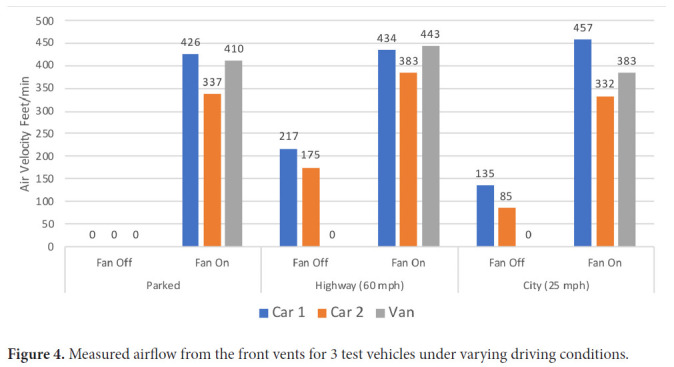
Measured airflow from the front vents for 3 test vehicles under varying driving conditions.

Figure 4 shows the measured airflow from the front vents for 2 cars (cars 1 and 2; the same cars 1 and 2 from Figures 1 and 3) and the van under varying driving conditions. No airflow was detected while the vehicles were parked with the fan off, whereas high flow rates were detected when the fans were on. Substantial airflow was detected when the fan was off in the cars during highway driving and to a lesser extent during city driving at 25 miles per hour; during city driving airflow was detected only while the vehicle was moving (ie, no airflow was detected while stopped at stop signs/traffic lights). The airflow measurements for car 1 while driving with the fan off were higher than for car 2; carbon dioxide levels rose above 800 ppm during highway driving with the fan off in car 2 but not in car 1 (Figure 3). No airflow was detected in the van while driving with the fan off, consistent with the finding that carbon dioxide levels rose to a much greater extent under these conditions in the van but not in the cars (Figure 3). For the van, similar airflow was detected for the side vents (data not shown).

## DISCUSSION

We found that carbon dioxide levels remained under 800 ppm in all vehicles tested if the fan was operated on non-recirculation mode or if 1 front window was partially or fully open. Carbon dioxide levels rose above 800 ppm in all motor vehicles during city driving or while parked if the windows were closed and the ventilation fan was off. During highway driving with the windows closed and fan off carbon dioxide levels rose above 800 ppm in 1 of 5 cars and in the van. The carbon dioxide was produced through natural respiration by vehicle occupants and the concentration was easily and rapidly measured with an inexpensive carbon dioxide meter (<$100).

Our findings suggest that carbon dioxide measurements could provide a simple, inexpensive, and rapid method to assess ventilation in motor vehicles. Previous studies have demonstrated the potential value of carbon dioxide monitoring as an indicator of ventilation and viral transmission risk in buildings [7–13]. Huang *et al* [9] found that air changes per hour varied considerably in dental procedure rooms, and carbon dioxide accumulated above 800 ppm in rooms with low ventilation (<6 air changes per hour) and overcrowding, but not in those with higher ventilation. Elevated carbon dioxide levels have not been directly linked to SARS-CoV-2 transmission risk, but poorly ventilated indoor spaces are generally considered high-risk areas [3, 8, 14]. According to the CDC, carbon dioxide monitoring can provide information on ventilation in indoor spaces, which can be used to enhance protection against COVID-19 transmission [13]. Carbon dioxide levels below 800 ppm were cited as a potential target benchmark for good ventilation to reduce the risk for viral transmission [13].

We found that carbon dioxide levels under differing ventilation conditions correlated well with measurement of airflow from vents allowing air entry for 3 vehicles where airflow was assessed (2 cars and the van). When the vehicles were parked with the fan off no airflow was detected from the vents and carbon dioxide levels rose. In contrast, when the fan was on while parked or when driving, high airflow (>300 feet/min) was detected, and carbon dioxide levels remained low. More modest airflow was detected during highway driving with the fan off in both cars (≥175 feet/min), but no airflow was detected under these conditions in the van; peak carbon dioxide levels were below 1,000 ppm in both cars, whereas the carbon dioxide level increased to nearly 1,500 ppm in the van. Even lower levels of airflow were detected in the cars during city driving at 25 miles per hour with the fan off (≤135 feet/min), and no airflow was detected when the cars were stopped at stop signs/traffic lights; during city driving carbon dioxide levels peaked above 1,100 ppm.

Our findings have several practical implications for efforts to improve ventilation to reduce the risk for transmission of SARS-CoV-2 when more than 1 occupant is in a motor vehicle. First, the ventilation system should be set on non-recirculation mode as recommended by the CDC [3]. Second, windows should be opened, or the fan should be operated when vehicles are parked or during city driving. Under these conditions, opening windows even partially or running the fan can markedly improve ventilation. Third, because ventilation may differ substantially in different vehicles, using a carbon dioxide monitor to measure carbon dioxide levels could be used to provide real-time feedback to improve ventilation. In schools, such feedback on carbon dioxide levels has been used to indicate when windows should be opened to improve classroom air quality [12].

Our study has some limitations. Only 6 motor vehicles were included in the study and carbon dioxide levels were monitored for relatively short periods. Additional studies are needed with a wider range of vehicles and with assessments during longer trips. In a recent report, transmission of SARS-CoV-2 from an infected driver to passengers in a van occurred despite physical distancing and continuous operation of the ventilation system [1]. Simulations demonstrated that airflow in the van facilitated transfer of fluorescent microspheres from the front to the back of the van [1]. Studies are needed to determine if interventions other than improved ventilation will be required to minimize viral transmission risk in motor vehicles. The CDC has recommended that barriers such as plexiglass shields be installed in work situations where distancing cannot be maintained [15–17]. Additional studies are needed to determine if such barriers could be useful in decreasing the risk for viral transmission in motor vehicles. In a simulation study, it was suggested that sneeze shields placed between passengers in an airplane could reduce the risk for aerosol transmission [16]. Finally, we cannot exclude the possibility that factors such as position of the vents or age of the vehicle might affect ventilation. However, similar results were obtained for each of the 6 study vehicles.

In summary, our findings suggest that carbon dioxide measurements could provide a simple, inexpensive, and rapid method to assess ventilation in motor vehicles. Studies are needed to determine if real-time carbon dioxide monitoring could be used to provide feedback to improve ventilation in motor vehicles and to reduce the risk for viral transmission.

## References

[1] Jones LD, Chan ER, Zabarsky TF, Cadnum JL, Navas ME, Redmond SN, Kovach JD, Linger M, Rutala WA, Zimmerman PA, Donskey CJ. Transmission of SARS-CoV-2 on a Patient Transport Van. *Clin Infect Dis.* 2021. doi: 10.1093/cid/ciab347. PubMed PMID: 33893474; PMCID: PMC8135457. PMC8135457 33893474

[2] Knibbs LD, Morawska L, Bell SC. The risk of airborne influenza transmission in passenger cars. *Epidemiol Infect.* 2012;140(3):474–8. doi: 10.1017/S0950268811000835. PubMed PMID: 21733264. 21733264

[3] Prevention CfDCa. Domestic Travel During COVID-19 Information for People Traveling within the United States and U.S. Territories 2022 [cited 2022 January 4]. Available from: https://www.cdc.gov/coronavirus/2019-ncov/travelers/travel-during-covid19.html.

[4] Shen Y, Li C, Dong H, Wang Z, Martinez L, Sun Z, Handel A, Chen Z, Chen E, Ebell MH, Wang F, Yi B, Wang H, Wang X, Wang A, Chen B, Qi Y, Liang L, Li Y, Ling F, Chen J, Xu G. Community Outbreak Investigation of SARS-CoV-2 Transmission Among Bus Riders in Eastern China. *JAMA Intern Med*. 2020;180(12):1665–71. doi: 10.1001/jamainternmed.2020.5225. PubMed PMID: 32870239; PMCID: PMC7489377. 32870239 PMC7489377

[5] Knibbs LD, de Dear RJ, Atkinson SE. Field study of air change and flow rate in six automobiles. *Indoor Air.* 2009;19(4):303–13. doi: 10.1111/j.1600-0668.2009.00593.x. PubMed PMID: 19500174. 19500174

[6] Mathai V, Das A, Bailey JA, Breuer K. Airflows inside passenger cars and implications for airborne disease transmission. *Sci Adv.* 2021;7(1). doi: 10.1126/sciadv.abe0166. PubMed PMID: 33277325; PMCID: PMC7775778. PMC7775778 33277325

[7] Batterman S. Review and Extension of CO(2)-Based Methods to Determine Ventilation Rates with Application to School Classrooms. *Int J Environ Res Public Health*. 2017;14(2). doi: 10.3390/ijerph14020145. PubMed PMID: 28165398; PMCID: PMC5334699. PMC5334699 28165398

[8] Ha W, Zabarsky TF, Eckstein EC, Alhmidi H, Jencson AL, Cadnum JL, Donskey CJ. Use of carbon dioxide measurements to assess ventilation in an acute care hospital. *Am J Infect Control*. 2021. doi: 10.1016/j.ajic.2021.11.017. PubMed PMID: 34848292; PMCID: PMC8627286. PMC8627286 34848292

[9] Huang Q, Marzouk T, Cirligeanu R, Malmstrom H, Eliav E, Ren YF. Ventilation Assessment by Carbon Dioxide Levels in Dental Treatment Rooms. *J Dent Res.* 2021;100(8):810–6. doi: 10.1177/00220345211014441. PubMed PMID: 33973494; PMCID: PMC8120146. 33973494 PMC8120146

[10] Schade W, Reimer V, Seipenbusch M, Willer U. Experimental Investigation of Aerosol and CO2 Dispersion for Evaluation of COVID-19 Infection Risk in a Concert Hall. *Int J Environ Res Public Health*. 2021;18(6). doi: 10.3390/ijerph18063037. PubMed PMID: 33809493; PMCID: PMC8002200. PMC8002200 33809493

[11] Villanueva F, Notario A, Cabanas B, Martin P, Salgado S, Gabriel MF. Assessment of CO2 and aerosol (PM2.5, PM10, UFP) concentrations during the reopening of schools in the COVID-19 pandemic: The case of a metropolitan area in Central-Southern Spain. *Environ Res.* 2021;197:111092. doi: 10.1016/j.envres.2021.111092. PubMed PMID: 33785326; PMCID: PMC8003457. 33785326 PMC8003457

[12] Wargocki P, Da Silva NA. Use of visual CO2 feedback as a retrofit solution for improving classroom air quality. *Indoor Air.* 2015;25(1):105–14. doi: 10.1111/ina.12119. PubMed PMID: 24735406. 24735406

[13] Prevention CfDCa. Ventilation in buildings 2021 [cited 2021 November 21]. Available from: https://www.cdc.gov/coronavirus/2019-ncov/community/ventilation.html.

[14] Lednicky JA, Lauzardo M, Alam MM, Elbadry MA, Stephenson CJ, Gibson JC, Morris JG, Jr. Isolation of SARS-CoV-2 from the air in a car driven by a COVID patient with mild illness. *Int J Infect Dis.* 2021;108:212–6. doi: 10.1016/j.ijid.2021.04.063. PubMed PMID: 33901650; PMCID: PMC8064821. 33901650 PMC8064821

[15] Cadnum JL, Jencson AL, Donskey CJ. Do plexiglass barriers reduce the risk for transmission of severe acute respiratory syndrome coronavirus 2 (SARS-CoV-2)? *Infect Control Hosp Epidemiol.* 2021:1–4. doi: 10.1017/ice.2021.383. PubMed PMID: 34726150. 34726150

[16] Talaat K, Abuhegazy M, Mahfoze OA, Anderoglu O, Poroseva SV. Simulation of aerosol transmission on a Boeing 737 airplane with intervention measures for COVID-19 mitigation. *Phys Fluids* (1994). 2021;33(3):033312. doi: 10.1063/5.0044720. PubMed PMID: 33897238; PMCID: PMC8060968. 33897238 PMC8060968

[17] Prevention CfDCa. Protecting workers: Guidance on mitigating and preventing the spread of COVID-19 in the workplace 2021 [cited 2021 November 21]. Available from: https://www.osha.gov/coronavirus/safework.

